# The role of comorbidities on periprocedural complications and outcomes in patients with defibrillators and cardiac resynchronization therapy: insights from the German device registry

**DOI:** 10.1007/s00392-025-02821-2

**Published:** 2025-12-22

**Authors:** Nina Becher, Matthias Hochadel, Jochen Senges, Lars Eckardt, Hüseyin Ince, Thomas Kleemann, Christoph Stellbrink, Johannes Brachmann, Werner Jung, Frederik Voss, Paulus Kirchhof, Tobias Toennis, Andreas Metzner

**Affiliations:** 1 Department of Cardiology, University Heart & Vascular Center Hamburg, University Medical Center Hamburg-Eppendorf, Martinistrasse 52, 20246 Hamburg, Germany; 2 German Center for Cardiovascular Research (DZHK), Partner Site Hamburg/Kiel/Luebeck, Hamburg, Germany; 3 Stiftung Institut Fuer Herzinfarktforschung (IHF), Ludwigshafen, Germany; 4 Clinic for Cardiology II - Electrophysiology, University Hospital Münster, Münster, Germany; 5 Department of Cardiology, Vivantes Klinikum Am Urban and Neukölln, Berlin, Germany; 6 Rostock University, Rostock, Germany; 7 Medizinische Klinik B, Klinikum Der Stadt Ludwigshafen gGmbH, Ludwigshafen Am Rhein, Germany; 8 Department of Cardiology, Klinikum Bielefeld Mitte, Bielefeld, Germany; 9 Department of Cardiology, Klinikum Coburg, Coburg, Germany; 10 Department of Cardiology, Schwarzwald-Baar Klinikum, Villingen-Schwenningen, Germany; 11 Innere Medizin III, Krankenhaus Der Barmherzigen Brüder, Trier, Germany; 12 Institute of Cardiovascular Sciences, University of Birmingham, Birmingham, UK

**Keywords:** Implantable cardioverter-defibrillator, Comorbidities, Comorbidity burden, Mortality

## Abstract

**Background:**

Cardiac implantable electronic devices (CIEDs) are increasingly implanted in older patients with multiple comorbidities. The impact of comorbidities on procedural complications and clinical outcomes during and after defibrillator implantation remains a subject of ongoing debate.

**Aim:**

To investigate the associations of the comorbidity burden on baseline characteristics, periprocedural complications, and on outcomes in patients with implantable cardioverter defibrillator (ICD) and cardiac resynchronization therapy with defibrillator (CRT-D) implantations or revisions.

**Methods:**

Patients who underwent ICD or CRT-D implantations or revisions at 50 centers were prospectively enrolled in the German Device Registry. Data on patient characteristics, periprocedural complications, and outcomes were collected. Patients were categorized into four groups based on cardiometabolic comorbidities (stroke, chronic kidney disease (CKD), diabetes, hypertension): group I (no comorbidities), group II (one), group III (two), and group IV (three or four). Primary outcomes included 1-year all-cause mortality, major adverse cardiac and cerebrovascular events (MACCE), and arrhythmic/non-arrhythmic events. The Kaplan–Meier analysis was used to determine 1-year mortality.

**Results:**

Overall, 5329 patients (mean age 65.2 years) underwent 3794 ICD and 1535 CRT-D implantations. Median follow-up was 17 months. Periprocedural complications (group I: 2.1%, group II: 1.5%, group III: 2.1%, group IV: 2.4%; *p* = 0.91) and in-hospital MACCE (group I: 0.2%, group II: 0.4%, group III: 0.6%, group IV: 0.4%; *p* = 0.25) were not related to comorbidity burden. Higher comorbidity burden was associated with a higher 1-year all-cause mortality (*p* < 0.001), but ICD shocks did not differ between groups (*p* = 0.97). The MADIT-ICD non-arrhythmic mortality score increased with comorbidities (*p* < 0.001), while the VT/VF score remained unchanged.

**Conclusions:**

Periprocedural complications do not appear to be affected by cardiometabolic comorbidities in patients undergoing ICD or CRT-D implantation in Germany. As expected, multimorbidity was associated with a higher risk of mortality and MACCE without detectable effects on ventricular arrhythmias.

**Graphical Abstract:**

The role of comorbidities on periprocedural complications and outcomes in patients with defibrillators and cardiac resynchronization therapy. Abbreviations: CD; comorbidity, CKD, chronic kidney disease; CRT-D/-P, cardiac resynchronization therapy-defibrillator/-pacemaker; ICD, implantable cardioverter-defibrillator; MACCE, major adverse cardiac and cerebrovascular events

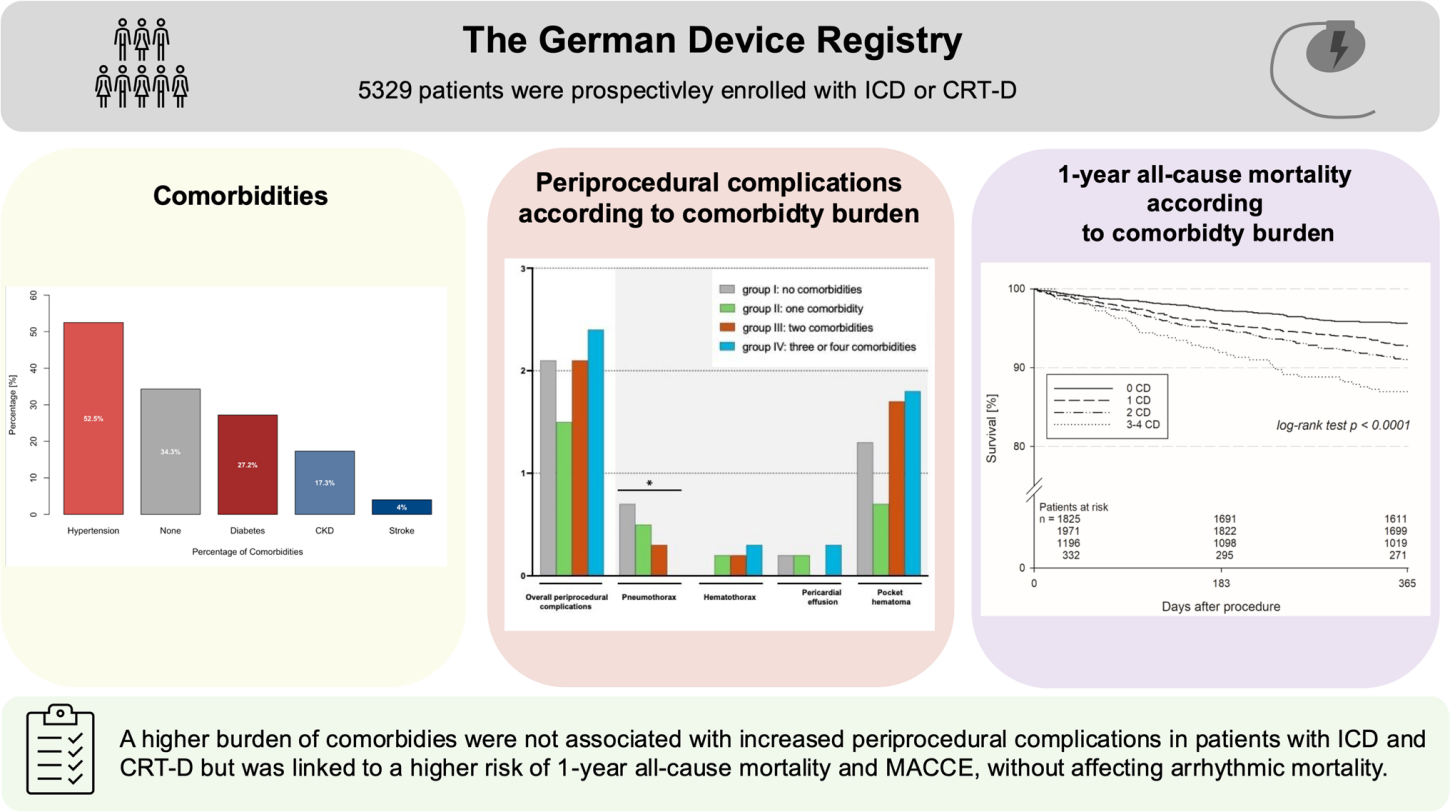

**Supplementary Information:**

The online version contains supplementary material available at 10.1007/s00392-025-02821-2.

## Introduction

The burden of comorbidities has gradually increased in patients with defibrillators due to the demographic shift towards an aging general population [1, 2]. To date, implantable cardioverter defibrillators (ICD) and cardiac resynchronization therapy defibrillator (CRT-D) have been established as integral components for primary and secondary prevention of arrhythmic death and treatment of heart failure [3, 4]. However, growing evidence suggests that the use of defibrillator therapy needs to be carefully weighed against the risk of periprocedural and long-term complications, life expectancy, and competing risks of non-arrhythmic death [1, 5, 6]. In particular, the preponderance of non-arrhythmic mortality over arrhythmic mortality has prompted discussions regarding the selection of patients for ICD therapy, especially in primary prevention [1, 6–8]. Additionally, several patients receiving therapy for secondary prevention have competing risks for non-arrhythmic death [1, 6, 8]. To accurately assess the prognosis of patients with defibrillators in the presence of multiple co-morbid conditions, it is essential to add evidence from large-scale prospective clinical registries, moving beyond the limitations of data derived from randomized controlled trials.

Therefore, in this study we aimed to investigate (1) the role of cardiometabolic comorbidities on periprocedural complications of ICD and CRT-D implantations or revisions and (2) to assess the role of comorbidity burden on outcomes such as 1-year all-cause mortality, major adverse cardiac and cerebrovascular events (MACCE), and arrhythmic and non-arrhythmic mortality and hospitalization.

## Methods

### Study population

The German Device Registry I and II was initiated as a prospective multicenter registry by the Institut für Herzinfarktforschung (IHF) (Ludwigshafen, Germany). Between March 2007 and February 2014, patients who underwent ICD or CRT-D were enrolled from 50 nationwide centers. The data was collected and managed by the IHF. After providing written informed consent, data was entered into a web-based electronic case report form by the participating centers. The study was conducted in accordance with the Declaration of Helsinki and approved by the local ethics committee.

Patient characteristics, (peri-)procedural data, device data, device indication, implantation procedures, periprocedural complications (pneumothorax, hemothorax, pericardial effusion, and pocket hematoma (all requiring intervention)), and history of comorbidities were collected at the time of the device implantation or device revision and provided by the participating centers.

Follow-up was conducted by a structured phone call by the IHF, which consisted of a questionnaire regarding cardiovascular and non-cardiovascular events, hospitalizations, arrhythmias, and ICD shocks, New York Heart Association (NYHA) class. In cases where patients could not be contacted by telephone, supplementary clinical information was obtained through their primary care physicians, consulting cardiologists, or local civil registry offices to determine vital and clinical status. In addition, follow-up data—including clinical assessments and device interrogations—were extracted from institutional medical records of the participating centers, provided that such documentation was available.

### Endpoints and statistical analysis

Outcomes of interest were 1-year all-cause mortality; arrhythmia events among survivors, such as resuscitation or ICD shock within 1 year; hospitalization; and periprocedural complications that required intervention. Major adverse cardiac and cerebrovascular events (MACCE) were defined as the occurrence of death (all-cause), myocardial infarction (MI), or stroke. Patients were categorized according to the number of cardiometabolic comorbidities including prior stroke, chronic kidney disease (CKD), diabetes, and arterial hypertension: group I: no comorbidities, group II: one comorbidity, group III: two comorbidities, group IV: three or four comorbidities. These categories were regarded as ordinal and compared by tests for trend. All-cause mortality and the combined event of death and ICD shock were analyzed as time-to-event data, while the other endpoints were analyzed as binary variables. Regression analyses adjusted for age (linear), sex, coronary artery disease, heart failure, secondary prevention, and atrial fibrillation were performed for the outcomes: logistic regression for non-fatal ICD shocks and periprocedural complications, and Cox regression for 1-year mortality.

The stability of the results was challenged (1) in a supplementary analysis, including further comorbidities as peripheral artery disease (PAD) and chronic obstructive pulmonary disease (COPD) (combining three to six comorbidities in group IV) and (2) in a sensitivity analysis weighting CKD with 2 points and the interaction of CKD and diabetes with 1 point.

The Multicenter Automatic Defibrillator Implantation Trial (MADIT)-ICD benefit score [6] was applied across the predefined patient subgroups according to comorbidity burden. The MADIT-ICD non-arrhythmic mortality score (death without experiencing sustained ventricular tachycardia (VT)/ventricular fibrillation (VF) at any time during follow-up) was calculated without including the body mass index in this cohort due to the high number of missing values in this registry.

Categorical variables are presented as percentages and absolute numbers and compared by the Cochran-Armitage test. Continuous variables are presented as median and interquartile range (IQR) and compared by the Jonckheere-Terpstra test. One-year (event-free) survival was estimated by the Kaplan–Meier method and compared between groups by the log-rank test for trend. All *p*-values are two-sided and not adjusted for multiple comparisons.

Statistical computations were performed using SAS version 9.4 (Cary, NC, USA).

## Results

### Patient characteristics

Overall, 5329 patients were included (mean age 65.2 years) with 3794 ICDs, 1535 CRT-D implants/revisions, with a median follow-up of 17 months. Two hundred fifteen (4.0%) patients had a history of stroke, 920 (17.3%) had CKD, 1448 (27.2%) had diabetes, 2800 (52.5%) had hypertension, and 1828 (34.3%) had none of these cardiometabolic comorbidities (see Graphical Abstract).

Detailed baseline characteristics according to the number of comorbidities are presented in Table 1. Patients with no comorbidities (group I) were more likely to be younger (group I: 63 years (interquartile range [IQR] 51, 72) than patients with more comorbidities (group II: 69 (60, 75), group III: 70 (63, 75), group IV: 72 (66, 76); *p* < 0.001). Patients with a higher burden of comorbidities had a higher NYHA class (*p* < 0.001) and suffered more frequently from ischemic cardiomyopathy (group I: 46.7%, group II: 64.9%, group III: 70.4%, group IV: 77.1%; *p* < 0.001). Patients with more comorbidities had more CRT-Ds than patients with fewer comorbidities (group I: 23.5%, group II: 28.6%, group III: 34.0%, group IV: 39.2%; *p* < 0.001). Regarding electrocardiogram parameters at baseline, patients with a higher burden of comorbidities more frequently presented with atrial fibrillation at baseline (group I: 14.1%, group II: 18.3%, group III: 23.9%, group IV: 26.2%; *p* < 0.001) and left bundle branch block (*p* < 0.001). No substantial differences in baseline characteristics were observed for patients with PAD or COPD (Supplementary Table S1).

**Table 1:** Baseline characteristics according to the number of comorbidities (CD) (groups I–IV)

30	Group I (no CD) (*N* = 1828)	Group II (one CD) (*N* = 1973)	Group III (two CD) (*N* = 1196)	Group IV (three or four CD) (*N* = 332)	*P*-value
Demographic data					
Age (years), *median*	63 (51; 72)	69 (60; 75)	70 (63; 75)	72 (66; 76)	< 0.001
Male (%)	79.3	81.1	83.4	79.8	0.049
BMI (kg/m^2^), *median*	25.9 (23.8; 28.8), *n* = 194	27.0 (24.6; 30.8), *n* = 260	27.9 (25.3; 31.3), *n* = 162	26.6 (23.8; 29.3), *n* = 52	< 0.001
NYHA classification in cardiac disease	(*n* = 1599)	(*n* = 1817)	(*n* = 1114)	(*n* = 313)	< 0.001
*NYHA I/none (%)*	23.8	17.0	11.0	6.7	
*NYHA II (%)*	37.9	40.3	38.7	33.9	
*NYHA III (%)*	35.9	40.6	45.6	52.7	
*NYHA IV (%)*	2.4	2.1	4.7	6.7	
*NYHA III* + *(%)*	38.3	42.7	50.3	59.4	< 0.001
LVEF (%)	30 (25; 40), *n* = 1742	30 (25; 35), *n* = 1895	30 (24; 34), *n* = 1158	28 (23; 33), *n* = 325	< 0.001
Cardiac disease					
Ischemic cardiomyopathy (%)	46.7	64.9	70.4	77.1	< 0.001
Prior myocardial infarction (%)	24.8	37.2	40.9	53.6	< 0.001
Dilated cardiomyopathy (%)	35.0	32.6	31.8	29.8	0.020
Hypertrophic cardiomyopathy (%)	5.4	2.4	1.8	1.2	< 0.001
Hypertensive heart disease (%)	2.1	7.5	10.4	10.8	< 0.001
Primary electric disease (%)	4.3	1.3	0.7	0	< 0.001
Type of device					
ICD (VVI) (%)	55.3	50.0	47.4	41.9	< 0,001
ICD (DDD) (%)	21.1	21.3	18.6	19.0	0.10
CRT-D (%)	23.7	28.6	34.0	39.2	< 0.001
ECG parameters at baseline					
Sinus rhythm (%)	83.1	77.7	72.9	69.6	< 0.001
Atrial fibrillation (%)	14.1	18.3	23.9	26.2	< 0.001
LBBB (%)	26.5	32.4	37.2	43.4	< 0.001
RBBB (%)	6.1	7.0	7,1	8,7	0,086
Other IVCD (%)	6.5	7.1	8,3	8.1	0.058
QRS (ms), median	110 (100; 144)	116 (100; 150)	120 (100; 152)	130 (104; 160)	< 0,001
Periprocedural characteristics					
ICD indication					
Primary prevention (%)	53.3	58.1	67.6	71.1	< 0.001
Secondary prevention (%)	46.7	41.9	32.4	28.9	< 0.001
*Ventricular fibrillation (%)*	43.3	38.7	35.6	33.3	0.002
*Ventricular tachycardia (%)*	41.8	45.0	50.8	50.0	0.003
*Syncope with inducible VT/VF (%)*	13.3	14.4	10.8	10.4	0.24
New implantations (%)	84.8	85.8	86.9	91.2	0.003
Revisions of an existing devices (%)	15.2	14.2	13.1	8.8	
Comorbidities					
Prior stroke (%)	0	2.5	7.6	22.6	–-
Diabetes (%)	0	16.7	67.3	94.6	–-
Arterial hypertension (%)	0	70.6	90.3	98.8	–-
Chronic kidney disease (%)	0	10.3	34.8	90.7	–-
*On haemodialysis (%)*	-	12.1	7.4	9.8	–-
Revascularization (PCI, CABG) (%)	29.1 (102/350)	45.1 (207/459)	51.3 (162/316)	55.6 (55/99)	< 0.001
Other cardiac surgery (%)	2.7	3.5	4.2	4.5	0.013
PAD (%)	1.1	2.7	5.4	8.7	< 0.001
COPD (%)	2.0	4.0	5.0	7.2	< 0.001

All variables are presented as mean ± SD or median and interquartile range or percentage. Completeness of documentation is > 99% except where stated otherwise

Abbreviations: *BMI* body mass index, *CABG* coronary artery bypass graft, *CD* comorbidity, *COPD* chronic obstructive pulmonary disease, *CRT-D* cardiac resynchronization therapy-defibrillator, *ICD* implantable cardioverter defibrillator, *ECG* electrocardiogram, *IVCD* intraventricular conduction delay, *LBBB* left bundle branch block, *LVEF* left ventricular ejection fraction, *NYHA* New York Heart Association, *PAD* peripheral artery disease, *PCI* percutaneous intervention, *RBBB* right bundle branch block, *VF* ventricular fibrillation, *VT* ventricular tachycardia

### Periprocedural and intrahospital complications

**Fig. 1:**
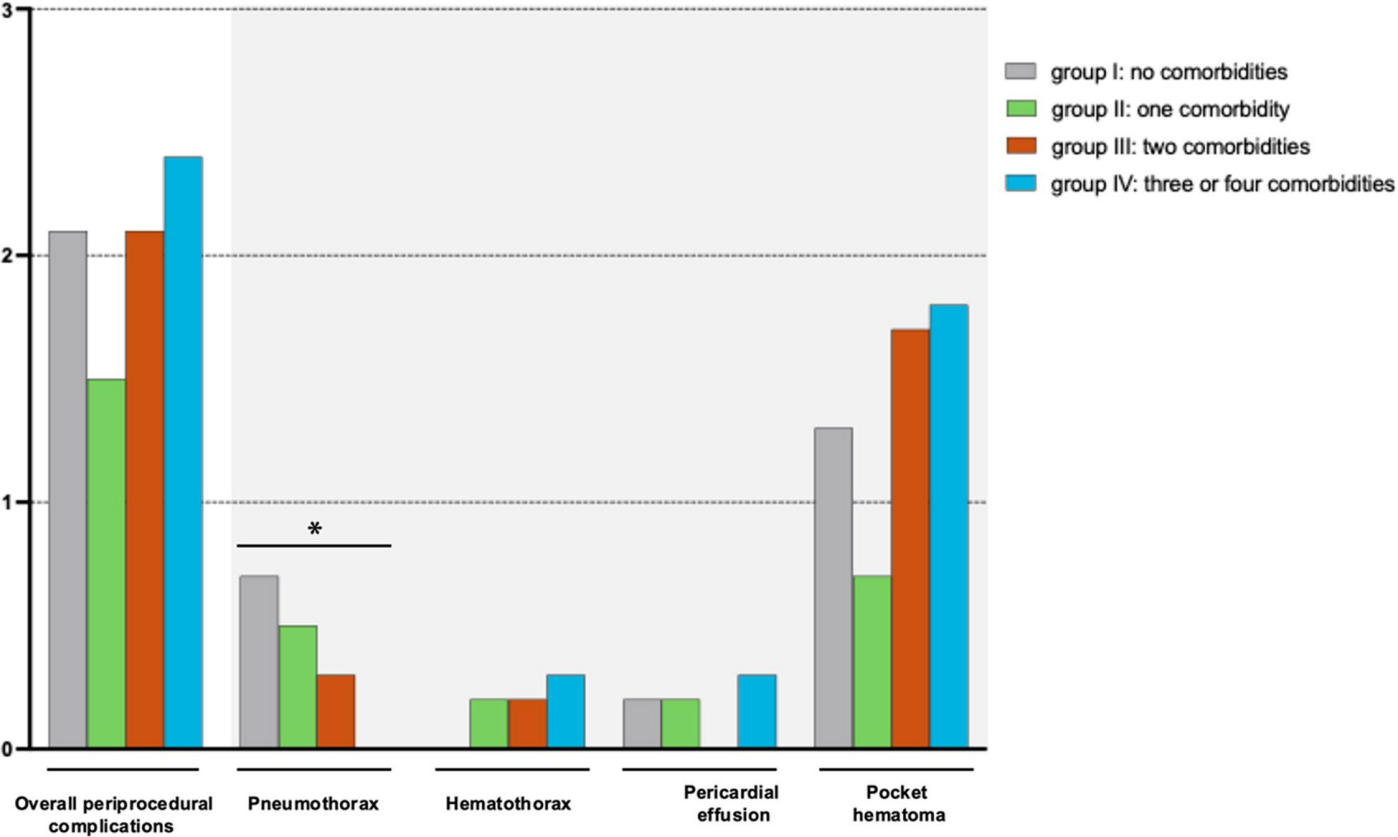
Periprocedural complications (needing interventions) according to the number of comorbidities (groups I–IV) including prior stroke, chronic kidney disease, diabetes, and arterial hypertension

Overall periprocedural complications (pneumothorax, hemothorax, pericardial effusion, and pocket hematoma) with the need for interventions, including ICD and CRT-D implantations and revisions, were not related to cardiometabolic comorbidity burden (group I: 2.1% (39/1822), group II: 1.5% (30/1962), group III: 2.1% (25/1186), group IV: 2.4% (8/331); *p* = 0.91) (Fig. 1 and Graphical Abstract).

A higher comorbidity burden in patients with ICD or CRT-D implantations or revisions was not associated with higher overall intrahospital complications (including periprocedural complications and in-hospital MACCE) (group I: 3.7% (54/1472), group II: 3.9% (59/1503), group III: 4.5% (39/872), group IV: 3.7% (9/232); *p* = 0.47) (Table 2). In-hospital MACCE occurred in 0.2% (3/1473) in patients with no comorbidities, in 0.4% (6/1508) in patients with one comorbidity, in 0.6% (5/876) in patients with two comorbidities, and 0.4% (1/233) in patients with three or four comorbidities (*p* = 0.25). In-hospital all-cause death was not associated with a higher comorbidity burden (*p* = 0.070) (Table 2). No significant differences were observed for patients with PAD or COPD (Supplementary Table S2).

**Table 2:** Overall intrahospital complications (including MACCE and periprocedural complications) and intrahospital MACCE (major adverse cardiac and cerebrovascular events)

	Group I (no CD) (***N*** = 1828)	Group II (one CD) (***N*** = 1973)	Group III (two CD) (***N*** = 1196)	Group IV (three or four CD) (***N*** = 332)	***P***-value
Intrahospital complications	3.7% (54/1472)	3.9% (59/1503)	4.5% (39/872)	3.9% (9/232)	0.47
MACCE intrahospital	0.2% (3/1473)	0.4% (6/1508)	0.6% (5/876)	0.4% (1/233)	0.25
*All-cause death*	0.1% (2/1826)	0.3% (6/1971)	0.6% (7/1196)	0.3% (1/332)	0.07
*Stroke*	0% (0/1471)	0.1% (1/1503)	0.1% (1/872)	0% (0/232)	0.44
*Myocardial infarction*	0.1% (1/1471)	0% (0/1503)	0% (0/872)	0% (0/232)	0.63

Abbreviations: *CD* comorbidity, *MACCE* major adverse cardiac and cerebrovascular events

**Fig. 2:**
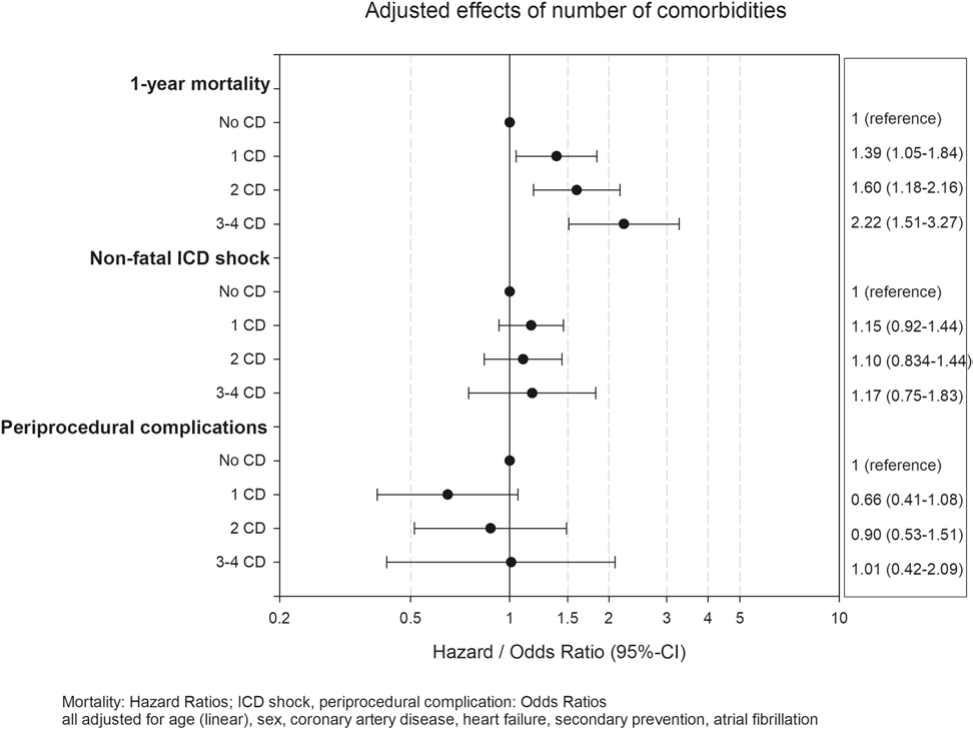
Adjusted analysis of clinical outcomes (1-year mortality, non-fatal ICD shock, periprocedural complications). Regression analyses were adjusted for age (linear), sex, coronary artery disease, heart failure, secondary prevention, and atrial fibrillation. Analyses were performed for the outcomes using logistic regression for non-fatal ICD shocks and periprocedural complications, and Cox regression for 1-year mortality

In the logistic regression analysis, no differences in the risk of periprocedural complications were observed across the comorbidity groups (Fig. 2).

### One-year follow-up: all-cause mortality, MACCE, and rehospitalization

**Fig. 3:**
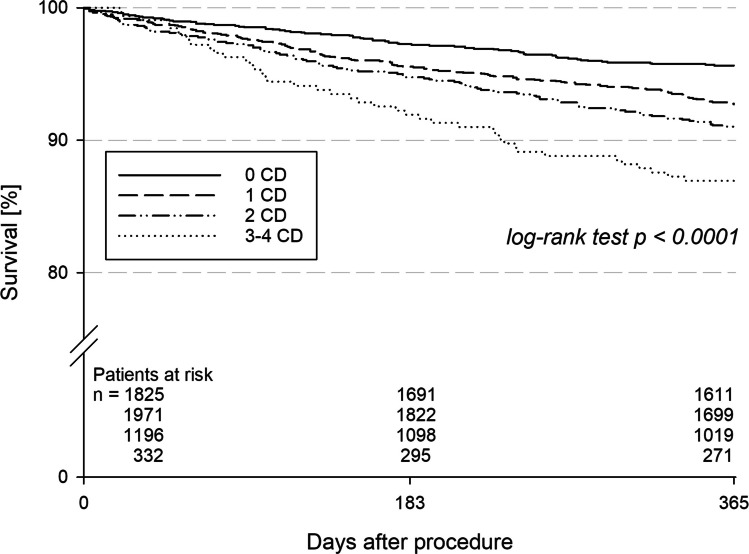
The Kaplan–Meier curves for 1-year all-cause mortality depending on number of comorbidities (CD) (groups I–IV: 0 CD; 1 CD; 2 CD; 3–4 CD) such as prior stroke, chronic kidney disease, diabetes, and arterial hypertension

Follow-up information could be obtained for 5154 (96.7%) of patients after a median follow-up period of 17 months. Follow-up of the events was analyzed at 365 days after ICD implantation. Patients with a higher burden of comorbidities had a higher risk for MACCE during 1 year (group I: 5.1% (90/1755), group II: 8.3% (158/1915), group III: 10.2% (119/1162), group IV: 14.9% (48/332); *p* < 0.001) (Table 3). A higher comorbidity burden was associated with a higher 1-year all-cause mortality (*p* < 0.001) (Fig. 3 and Table 3). Rehospitalization and duration of in-hospital stay were associated with a higher comorbidity burden (group I: 26.6% (361/1357), group II: 27.8% (401/1443), group III: 31.8% (262/823), group IV: 37.3% (78/209); *p* < 0.001), but not device-associated hospitalization (*p* = 0.65). ICD shocks did not differ among the groups (group I: 12.7% (178/1398), group II: 13.9% (206/1478), group III: 12.7% (109/856), group IV: 12.8% (28/219); *p* = 0.97) (Table 3). Comparable results were observed in the subgroup of patients undergoing ICD implantation for primary prevention only (Table 4).

**Table 3:** Arrhythmic risk and non-arrhythmic mortality risk for all patients (primary and secondary prevention) and events after implantation after one year follow-up

	Group I (no CD)	Group II (one CD)	Group III (two CD)	Group IV (three or four CD)	***P***-value
MADIT-ICD VT/VF-Score	7 (5; 8)	7 (5; 8)	7 (5; 8)	7 (5; 9)	0.21
VT/VF-Score ≥ 7	51.0% (350/686)	51.9% (405/780)	51.3% (270/526)	53.1% (78/147)	0.74
MADIT-ICD nonarrhythmic mortality Score*	2 (1; 3)	2 (1; 3)	3 (2; 4)	3 (2; 5)	< 0.001
Mortality-Score ≥ 3	36.6% (501/1370)	45.5% (695/1529)	59.7% (592/992)	71.6% (209/292)	< 0.001
*Events after implantation*					
ICD shocks	12.7% (178/1398)	13.9% (206/1478)	12.7% (109/856)	12.8% (28/219)	0.97
1-year mortality	4.4%	7.3%	9.0%	13.1%	< 0.001
Death/ICD shock	14.5%	18.0%	18.4%	21.8%	< 0.001
VT or incessant VT	1.6% (22/1340)	1.4% (20/1422)	2.0% (16/797)	0% (0/207)	0.55
Hospitalization	26.6% (361/1357)	27.8% (401/1443)	31.8% (262/823)	37.3% (78/209)	< 0.001
Resuscitation	0.6% (8/1446)	0.4% (6/1525)	0.6% (5/886)	0% (0/229)	0.51
MACCE	5.1% (90/1755)	8.3% (158/1915)	10.2% (119/1162)	14.9% (48/322)	< 0.001

Abbreviations: *ICD* implantable cardioverter defibrillator, *MACCE* major adverse cardiac and cerebrovascular events, *MADIT* Multicenter Automatic Defibrillator Implantation Trial, *VF* ventricular fibrillation, *VT* ventricular tachycardia

^*^without body mass index

**Table 4:** Arrhythmic risk and non-arrhythmic mortality risk for patients with indication for primary prevention and events after implantation for patients after 1-year follow-up

	Group I (no CD)	Group II (one CD)	Group III (two CD)	Group IV (three or four CD)	***P***-value
MADIT-ICD VT/VF-Score	6 (5; 8)	7 (5; 8)	7 (5; 8)	7 (5; 9)	0.10
VTVF-Score ≥ 7	49.0% (203/414)	50.7% (265/523)	51.0% (199/390)	52.3% (58/111)	0.49
MADIT-ICD nonarrhythmic mortality Score*	2 (1; 3)	2 (1; 3)	3 (2; 4)	3 (2; 5)	< 0.001
Mortality-Score ≥ 3	40.4% (326/806)	47.3% (449/950)	61.4% (431/702)	72.4% (152/210)	< 0.001
*Events after implantation*					
ICD shocks	10.5% (79/755)	11.7% (101/866)	11.0% (66/601)	11.4% (19/167)	0.72
1-year mortality	4.2%	5.5%	7.9%	10.5%	< 0.001
Death/ICD shock	12.7%	14.6%	16.4%	18.8%	0.005
VT or incessant VT	1.6% (11/708)	1.5% (12/815)	1.6% (9/549)	0% (0/157)	0.41
Hospitalization	27.6% (204/739)	28.2% (239/848)	31.8% (184/579)	37.6% (59/157)	0.009
Resuscitation	0.6% (5/780)	0.1% (1/902)	0.3% (2/618)	0.3% (2/618)	0.16
MACCE	4.9% (46/933)	8.9% (70/788)	8.9% (70/788)	12.2% (28/230)	< 0.001

^*^without body mass index

Abbreviations: *ICD* implantable cardioverter defibrillator, *MACCE* major adverse cardiac and cerebrovascular events, *MADIT* Multicenter Automatic Defibrillator Implantation Trial, *VF* ventricular fibrillation, *VT* ventricular tachycardia

In Cox regression analysis, a higher comorbidity burden was associated with a greater risk of 1-year mortality, showing a continuous increase with the number of comorbidities (Fig. 2). No differences were observed in the sensitivity analyses, both in the model including COPD and PAD as additional comorbidities (Supplementary Figure S1) and in the model applying weighted scoring for CKD and the combination of CKD and diabetes (Supplementary Fig S2). 

### Arrhythmic risk and non-arrhythmic mortality risk

The MADIT-ICD-Benefit VT/VF Score [6] for all patients (primary and secondary prevention) was 5 (7; 9) points and did not differ between the groups (*p* = 0.21) (Table 3). The MADIT-ICD non-arrhythmic mortality score was higher in patients with a higher comorbidity burden (group I: 2 (1; 3), group II: 2 (1; 3), group III: 3 (2; 4), group IV: 3 (2; 4) (*p* < 0.001). MADIT-ICD non-arrhythmic mortality score ≥ 3 was highest in patients with the highest comorbidity burden for all patients (group I: 36.6%, group II: 45.4%, group III: 59.6%, group IV: 71.6% (*p* < 0.001) (Table 3). After 1-year follow-up, non-lethal ICD shocks did not differ significantly between the study groups for all patients (primary and secondary prevention) (group I: 12.7%, group II: 13.9%, group III: 12.7%, group IV: 12.8% (*p* = 0.97)).

In patients who received a defibrillator solely for primary prevention, the non-arrhythmic score increased with the number of comorbidities, whereas the VT/VF score remained unchanged (Table 4). The risk of non-lethal ICD shocks in the primary prevention subgroup did not differ significantly between the groups after 1 year (group I: 10.5%, group II: 11.7%, group III: 11.0%, group IV: 11.4% (*p* = 0.72).

In a supplementary analysis, comorbidities such as PAD and COPD were included. The MADIT-ICD Benefit VT/VF Score was similar across these groups (Supplementary Table S3).

## Discussion

In this large-scale prospective German Device Registry, a higher burden of cardiometabolic comorbidities was not associated with increased periprocedural complications in patients with an ICD or CRT-D implantation or revision. As expected, more cardiometabolic comorbidities were associated with a higher 1-year all-cause mortality and MACCE and hospitalization. We found that a higher comorbidity burden was associated with a higher risk of non-arrhythmic mortality but not with arrhythmic death or ICD shocks. Thus, our study highlights the importance of a higher comorbid burden, including prior stroke, CKD, diabetes, and arterial hypertension, in weighing the risk of arrhythmic and non-arrhythmic risk of death in ICD and CRT-D patients.

Previous analyses from the German Device Registry evaluated the impact of individual comorbidities such as diabetes and chronic kidney disease on periprocedural complications and 1-year MACCE [9]. Our study expands on these findings by assessing the combined burden of multiple cardiometabolic comorbidities and incorporating the MADIT-ICD risk score and offering a more detailed view specifically for the subgroup of patients receiving devices for primary prevention.

In our study, the overall intra-hospital complications (3.7–4.5%), periprocedural major complications such as pneumothorax, hematothorax, pericardial effusion, and pocket hematoma with need for intervention (1.5–2.4%), and intrahospital MACCE (0.2–0.6%) did not differ among comorbidity burdens and were comparable to other studies [10]. In contrast, prior studies in patients with ICD or CRT implantations reported a higher risk of ~ 10% periprocedural complications [11]. Differences in reported periprocedural complication rates may be attributable to variants of reporting across registries and studies [10, 11].

In randomized controlled clinical trials, specific exclusion criteria, the consent and enrollment procedures, and the potential for therapy with a novel intervention or device can lead to the selection of healthier patients, leading to an underrepresentation or exclusion of patients with a high rate of comorbidities [12–16]. Additionally, these trials may have underestimated comorbidities as a competing risk [17]. Analyses of data sets reflecting routine care and observational research projects provide more information on patients with multiple comorbidities. The main finding in this study is that the implantation of devices does not lead to more complications in patients with cardiometabolic comorbidities. This supports current guidelines recommending that the decision for device implantation should balance the benefit of device protection against the competing risk of death and factors that may affect quality of life [3]. These findings align with our study, which showed that a higher comorbidity burden—such as prior stroke, CKD, diabetes, and arterial hypertension—in patients undergoing ICD or CRT implantation or revisions was associated with a higher risk for 1-year all-cause mortality and MACCE [1, 2, 7, 8, 18]. Moreover, similar to our findings in an earlier meta-analysis, patients in our study had a higher rate of hospitalizations during follow-up [7].

Personalized risk assessment is desirable because ICDs are highly effective in preventing sudden cardiac death due to arrhythmic events, but they do not prevent other forms of death, such as those resulting from heart failure or other comorbidities [8]. The MADIT-ICD benefit score was introduced as a clinical tool to evaluate patients ‘ specific risk for primary prevention of arrhythmic events such as VT/VF with the competing risk of non-arrhythmic mortality [6]. Interestingly, in the MADIT-ICD benefit, CKD was not considered in the score although it has been shown to be associated with reduced survival despite appropriate ICD therapies [8, 19, 20]. Our study demonstrated that comorbidities such as prior stroke, CKD, diabetes, and arterial hypertension—and importantly, the comorbidity burden—influenced the risk of non-arrhythmic mortality, although the VT/VF score did not differ across the groups. Similar to our study, in the Danish nationwide register, a higher comorbidity burden was not associated with a higher likelihood of appropriate ICD therapies but with a higher all-cause mortality (> 50% mortality risk at 4 years in patients with ≥ 3 comorbidities) [1]. Interestingly, in our study, we found no association between increasing comorbidity burden and the risk of ICD shock. The observed increase in mortality with a higher number of comorbidities appears to be primarily driven by non-arrhythmic causes of death. This is supported by the MADIT score as well as by the unchanged frequency of non-fatal ICD shocks. Moreover, in the future, arrhythmic risk estimation will probably require consideration of other factors, e.g., subclassification of non-ischemic dilated cardiomyopathies [21], ECG parameters including the use of artificial intelligence or deep neural networks [22, 23], genetics [24], biomarker imaging techniques such as magnetic resonance imaging [25], and left ventricular ejection fraction or other factors [26, 27]. However, in patients with prior ischemic cardiomyopathy, left ventricular ejection fraction and other predictors had poor predictive performance for sudden cardiac death, making accurate risk stratification and identification of candidates for defibrillator protection unfeasible [28].

The number of patients that must be treated (NNT) to justify especially primary prevention ICD [29] implantation remains uncertain and depends on factors such as the baseline risk of sudden cardiac death (SCD) within the patient population, the ICD’s effectiveness in lowering SCD risk, and competing risks, including non-arrhythmic mortality from other comorbidities. Our study may help to refine ICD therapy selection for those who are most likely to benefit.

Contextualizing our findings within the study period is important, as heart failure treatment strategies have evolved substantially since then. This prospective study was conducted between March 2007 and February 2014 and therefore does not include patients receiving contemporary heart failure therapies or reflect current guideline recommendations [4, 30]. Contemporary guideline-directed quadruple therapy, which includes angiotensin receptor-neprilysin inhibitors (ARNI), beta-blockers, mineralocorticoid receptor antagonists, and (sodium-glucose cotransporter-2) SGLT2, has substantially improved outcomes in ICD and CRT-D populations across both primary and secondary prevention indications [31–34]. By reducing HF progression, non-arrhythmic mortality, and cardiovascular death, and with emerging evidence suggesting fewer arrhythmic events and sudden cardiac death [35], modern therapy may lower the overall need for appropriate ICD interventions in current clinical practice. As these treatments mitigate systemic and heart failure-related risk pathways, the prognostic role of comorbidity burden, and therefore the risk gradients observed in our cohort, which was enrolled before the widespread adoption of ARNI and SGLT2 inhibitors, may be less pronounced in optimally treated contemporary ICD and CRT-D patients.

Despite these therapeutic advances, our dataset provides an important real-world reference point, capturing the natural risk profile and device-related outcomes in the era preceding contemporary quadruple therapy. Moreover, persistent therapeutic inertia, including the underuse and underdosing of guideline-directed treatment, particularly in patients with chronic kidney disease or polypharmacy, remains highly prevalent [36–38] and indicates that many contemporary ICD and CRT-D recipients still resemble our study population with respect to treatment exposure and residual risk.

## Limitations

The study has several limitations that have to be considered when interpreting the results. Data acquisition is variable according to the data entry of the participating centers. The low prevalence of chronic obstructive pulmonary disease (COPD) and peripheral artery disease (PAD), likely influenced by diagnostic difficulties and symptom overlap, especially in heart failure patients, and the fact that malignancies were not captured, limited their inclusion in our primary analyses. The four selected cardiometabolic comorbidities are well-recognized, strong predictors of adverse outcomes in this population; nevertheless, the exclusion of malignancies (not captured in the registry) could have influenced our findings, especially regarding outcomes. Due to the registry nature of the study, there is a risk of selection bias and confounding Residual or unmeasured confounding may remain and should be considered when interpreting our results. The MADIT-ICD non-arrhythmic mortality score was applied in a modified form excluding BMI due to missing data, which may have impacted risk estimation. Reliance on telephone follow-up and site-reported data without central adjudication introduces a potential risk of misclassification, for example, by not distinguishing between appropriate and inappropriate ICD shocks. Furthermore, the possibility of underreporting of events cannot be excluded.

The study was conducted between March 2007 and February 2014 and therefore does not include patients receiving contemporary heart failure therapies or fully reflect current guideline-directed care. As such, the applicability of our findings to current clinical practice in 2025 is limited. Furthermore, because the data were derived from a German registry, the results may not be entirely generalizable to other populations**.**

## Conclusions

In the prospective German Device Registry including patients with ICD and CRT-D implantations and revisions, a higher comorbidity burden was not associated with an increased risk of periprocedural complications. Importantly, a higher burden of cardiometabolic comorbidities was associated with an elevated risk of 1-year all-cause mortality, MACCE, hospitalizations, and with a higher risk of non-arrhythmic mortality. The increase in all-cause mortality with a higher number of comorbidities appears to be primarily driven by non-arrhythmic causes of death. This is supported by the MADIT score and further corroborated by the consistent rate of non-fatal ICD shocks across all comorbidity groups. The presence of competing comorbidities should be considered when evaluating the long-term benefits of CIED therapy. Further studies are warranted to evaluate a more personalized approach for risk stratification in patients with concomitant comorbidities undergoing ICD and CRT-D implantations.

## Supplementary Information

Below is the link to the electronic supplementary material.

Supplementary file1 (DOCX 2302 KB)

## Data Availability

The data supporting this study are curated at the Foundation Institut für Herzinfarktforschung, Ludwigshafen, Germany. The data are not publicly available but may be obtained from the corresponding authors upon reasonable request.
