# Supplementary material for: The role of comorbidities on periprocedural complications and outcomes in patients with defibrillators and cardiac resynchronization therapy: insights from the German device registry
Source: Clin Res Cardiol. 2025 Dec 22;115(4):645–56. doi: 10.1007/s00392-025-02821-2 (PMC13013360; doi:10.1007/s00392-025-02821-2)
Supplement: Supplementary file 1 — Supplementary file1 (DOCX 2302 KB) [file 392_2025_2821_MOESM1_ESM.docx]

**Supplementary Material**

Supplementary Material to: Becher N et al. The role of Comorbidities on Periprocedural Complications and Outcomes in Patients with Defibrillators and Cardiac Resynchronization Therapy: Insights from the German Device Registry

**Table of Contents**   **Page number**

**Supplementary Table**

Supplementary Table S1. 2

Supplementary Table S2. 3

Supplementary Table S3. 4

Supplementary Figure S1. 5

Supplementary Figure S2. 6

**Table S1.** Baseline characteristics according to the number of comorbidities (CD) (group I-IV). Comorbidities included prior stroke, chronic kidney disease, diabetes and arterial hypertension. peripheral artery disease (PAD) and chronic obstructive pulmonary disease (COPD).

|  | **Group I**  **(no CD)**  **(N=1776)** | **Group II**  **(one CD)**  **(N= 1896)** | **Group III**  **(two CD)**  **(N=1204)** | **Group IV**  **(three or six CD)**  **(N=453)** | **P-value** |
| --- | --- | --- | --- | --- | --- |
| **Demographic data** |  |  |  |  |  |
| Age (years) , *median* | 63 (51; 72) | 68 (60; 74) | 70 (63; 75) | 72 (65; 76) | < 0.001 |
| Male (%) | 79.1 | 81.1 | 83.2 | 81.2 | 0.021 |
| *NYHA III+ (%)* | 37.8 | 42.0 | 50.4 | 58.8 | < 0.001 |
| LVEF ≤ 30% (%) | 55.4 | 61.1 | 61.1 | 72.3 | < 0.001 |
| **Cardiac disease** |  |  |  |  |  |
| Coronary heart disease (%) | 45.9 | 64.8 | 69.1 | 78.4 | < 0.001 |
| Prior myocardial infarction (%) | 24.5 | 37.1 | 40.5 | 50.3 | < 0.001 |
| Dilated cardiomyopathy (%) | 34.6 | 33.0 | 32.1 | 30.0 | 0.037 |
| Hypertrophic cardiomyopathy (%) | 5.6 | 2.4 | 1.8 | 1.1 | < 0.001 |
| Hypertensive Heart Disease (%) | 2.0 | 7.6 | 9.6 | 11.0 | < 0.001 |
| Primary electric disease (%) | 4.4 | 1.4 | 0.6 | 0.2 | < 0.001 |
| **Type of device** |  |  |  |  |  |
| ICD (VVI) (%) | 55.2 | 50.3 | 50.3 | 45.0 | < 0.001 |
| ICD (DDD) (%) | 21.2 | 21.5 | 19.0 | 17.2 | 0.030 |
| CRT-D (%) | 23.5 | 28.3 | 34.1 | 37.7 | < 0.001 |
| **ECG parameters at baseline** |  |  |  |  |  |
| Atrial fibrillation (%) | 14.1 | 18.3 | 23.3 | 26.25 | <0.001 |
| **Periprocedural characteristics** |  |  |  |  |  |
| **ICD indication** |  |  |  |  |  |
| Primary prevention (%) | 52.7 | 58.1 | 66.5 | 72.2 | <0.001 |
| Secondary prevention (%) | 47.3 | 41.9 | 33.5 | 27.8 | <0.001 |

All variables are presented as mean ± SD or median and interquartile range or percentage. Completeness of documentation is > 99% except where stated otherwise. **Abbreviations:** CD**,** comorbidity; COPD, chronic obstructive pulmonary disease; CRT-D, cardiac resynchronization therapy-defibrillator; ICD, implantable cardioverter defibrillator; ECG; electrocardiogram; LVEF, left ventricular ejection fraction; NYHA, New York Heart Association; PAD, peripheral artery disease

**Table S2.** Overall intrahospital complications (Periprocedural complications and intrahospital MACCE (major adverse cardiac and cerebrovascular events) and events after one year (1-year mortality, ICD shocks, hospitalization). Comorbidities included prior stroke, chronic kidney disease, diabetes and arterial hypertension, peripheral artery disease (PAD) and chronic obstructive pulmonary disease (COPD).

|  | **Group I**  **(no CD)**  **(N=1828)** | **Group II**  **(one CD)**  **(N= 1973)** | **Group III**  **(two CD)**  **(N=1196)** | **Group IV**  **(three or six CD)**  **(N=332)** | **P-value** |
| --- | --- | --- | --- | --- | --- |
| **Periprocedural complications^a^** | 2.1% (37/1770) | 1.6% (31/1886) | 2.1% (25/1194) | 2.0% (9/451) | 0.98 |
| **MACCE intrahospital** | 0.2% (3/1448) | 0.3 % (5/1468) | 0.7% (6/891) | 0.4% (1/283) | 0.16 |
| **1-year mortality** | 4.3% | 6.9% | 8.6% | 14.0% | < 0.001 |
| **ICD shocks** | 12.7% (174/1367) | 13.9% (201/1441) | 12.9 % (114/883) | 11.6 % (35/301) | 0.78 |
| **Hospitalization** | 39.6% (526/1328) | 39.6% (555/1403) | 46.3% (386/834) | 47.0% (132/281) | < 0.001 |

^a^ Periprocedural complications included pneumothorax, hemothorax, pericardial effusion, and pocket hematoma, all requiring intervention.

**Abbreviations:** CD, comorbidity; MACCE, major adverse cardiac and cerebrovascular events

**Table S3.** Arrhythmic risk and non-arrhythmic mortality risk for all patients (primary and secondary prevention). Comorbidities included prior stroke, chronic kidney disease, diabetes and arterial hypertension, peripheral artery disease (PAD) and chronic obstructive pulmonary disease (COPD).

|  | **Group I (no CD)** | **Group II**  **(one CD)** | **Group III**  **(two CD)** | **Group IV (three or four CD)** | **P-Value** |
| --- | --- | --- | --- | --- | --- |
| **MADIT-ICD VT/VF-Score** | 7 (5; 8) | 7 (5; 8) | 7 (5; 8) | 7 (5; 8) | 0.50 |
| **VT/VF-Score ≥ 7** | 50.5% (326/646) | 51.8% (367/708) | 50.8% (270/532) | 55.8 % (144/258) | 0.29 |
| **MADIT-ICD nonarrhythmic mortality Score*** | 2 (1; 3) | 2 (1; 3) | 3 (2; 4) | 3 (2; 5) | <0.001 |
| **Mortality-Score ≥ 3** | 36.3% (484/1333) | 45.2% (664/1469) | 58.4 % (580/994) | 68.7 % (272/396) | < 0.001 |

*without body mass index

**Abbreviations:** ICD, implantable cardioverter defibrillator; MADIT, Multicenter Automatic Defibrillator Implantation Trial, VF, ventricular fibrillation; VT, ventricular tachycardia

**Figure S1.** Adjusted sensitivity analysis of clinical outcomes (1-year mortality, non-fatal ICD shock, periprocedural complications) by number of comorbidities including PAD and COPD. Regression analyses were adjusted for age (linear), sex, coronary artery disease, heart failure, secondary prevention and atrial fibrillation were performed for the outcomes, by logistic regression for non-fatal ICD shocks and periprocedural complications, by Cox regression for 1-year mortality.


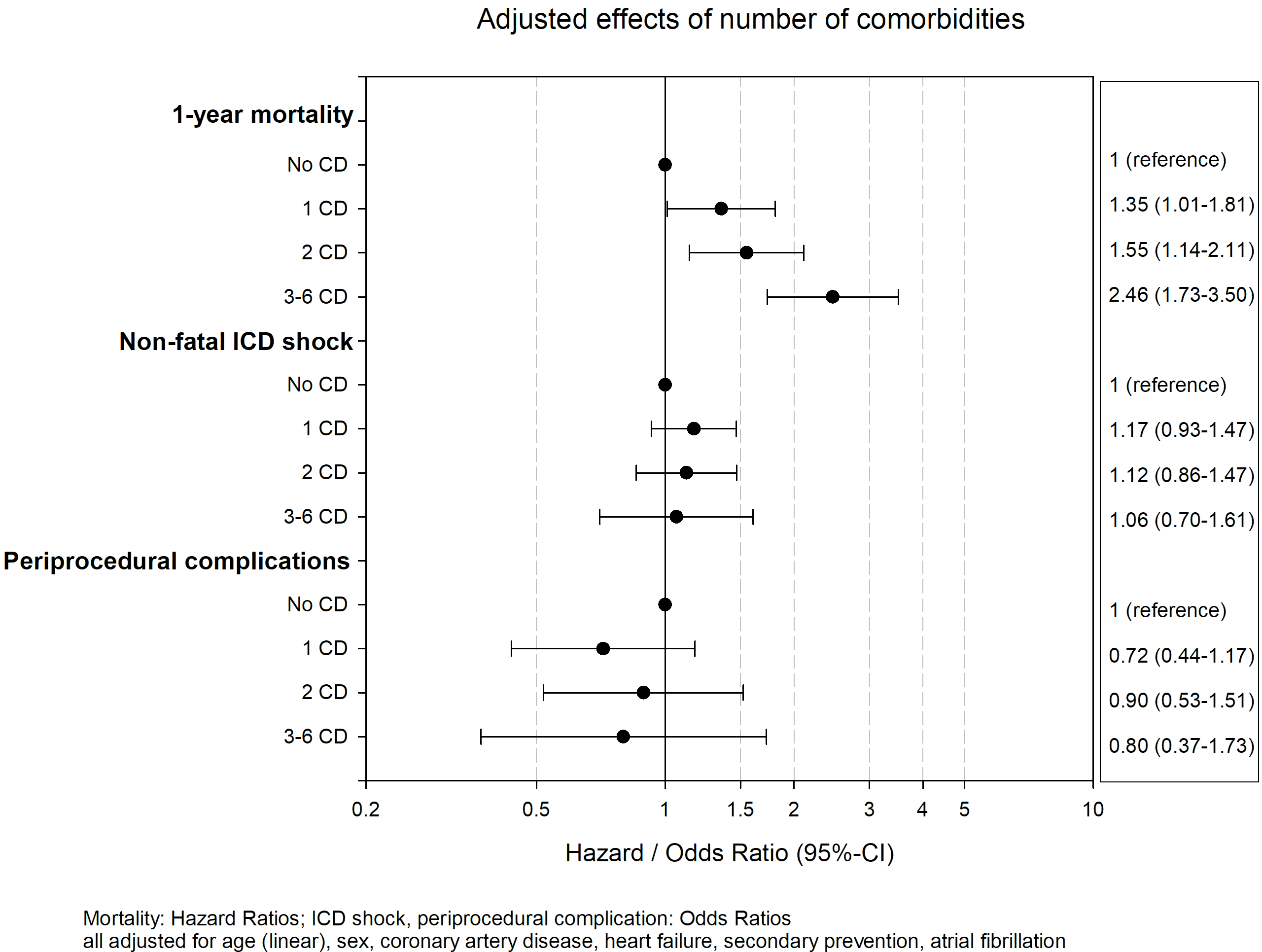


**Figure S2.** Adjusted sensitivity analysis of clinical outcomes (1-year mortality, non-fatal ICD shock, periprocedural complications) by comorbidities. CKD was weighted with 2 points and the interaction between CKD and diabetes with 1 point. Regression analyses were adjusted for age (linear), sex, coronary artery disease, heart failure, secondary prevention and atrial fibrillation were performed for the outcomes, by logistic regression for non-fatal ICD shocks and periprocedural complications, by Cox regression for 1-year mortality.

*
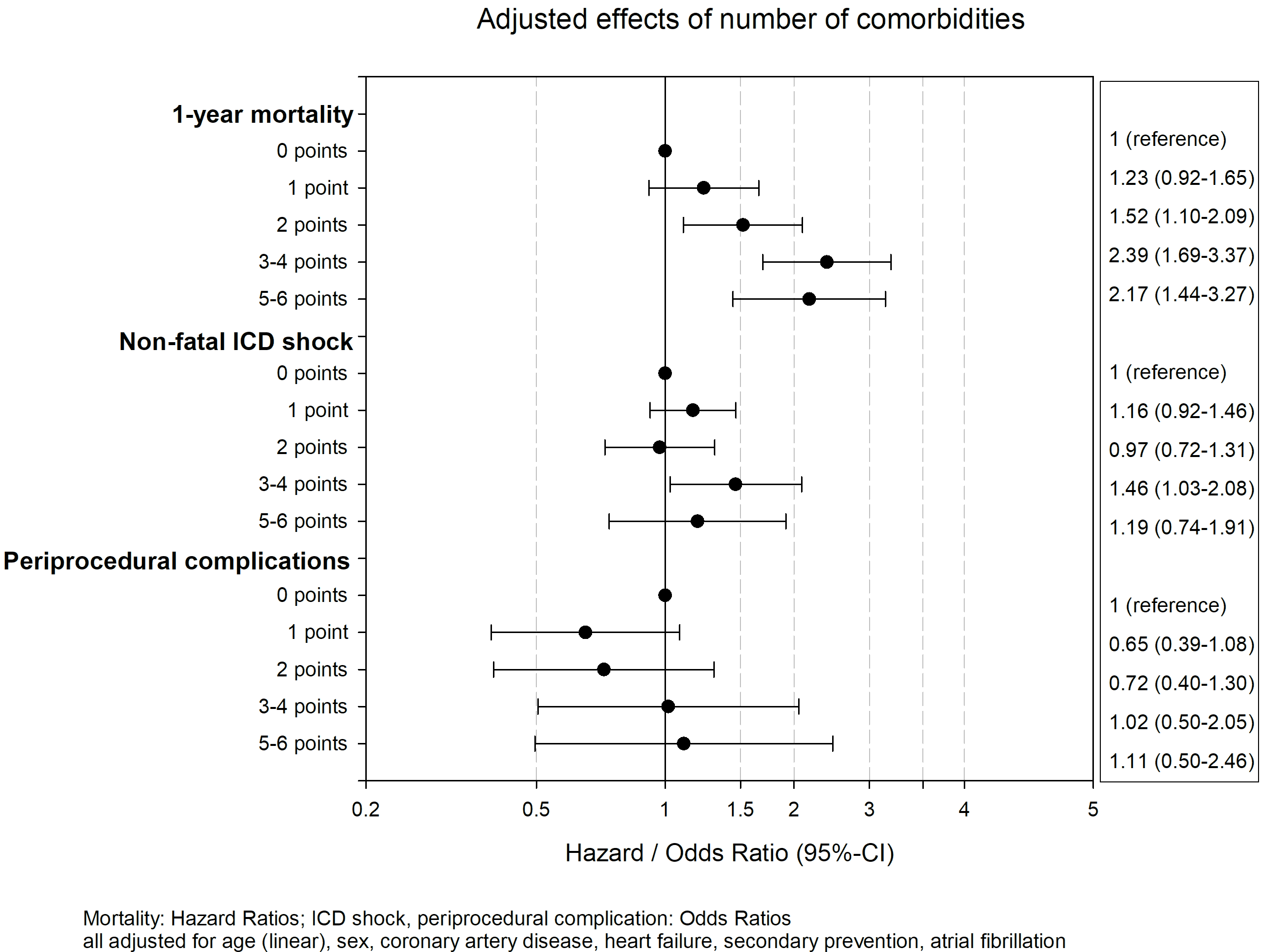
*
